# Identification of schizophrenia symptom-related gene modules by postmortem brain transcriptome analysis

**DOI:** 10.1038/s41398-023-02449-8

**Published:** 2023-05-04

**Authors:** Kazusa Miyahara, Mizuki Hino, Risa Shishido, Atsuko Nagaoka, Ryuta Izumi, Hideki Hayashi, Akiyoshi Kakita, Hirooki Yabe, Hiroaki Tomita, Yasuto Kunii

**Affiliations:** 1 Department of Disaster Psychiatry, International Research Institute of Disaster Science, Tohoku University, Sendai, Japan; 2 Department of Neuropsychiatry, School of Medicine, Fukushima Medical University, Fukushima, Japan; 3 Department of Pathology, Brain Research Institute, Niigata University, Niigata, Japan; 4 Department of Psychiatry, Tohoku University Hospital, Miyagi, Japan; 5 Department of Psychiatry, Graduate School of Medicine, Tohoku University, Miyagi, Japan

**Keywords:** Molecular neuroscience, Clinical genetics, Medical genetics

## Abstract

Schizophrenia is a multifactorial disorder, the genetic architecture of which remains unclear. Although many studies have examined the etiology of schizophrenia, the gene sets that contribute to its symptoms have not been fully investigated. In this study, we aimed to identify each gene set associated with corresponding symptoms of schizophrenia using the postmortem brains of 26 patients with schizophrenia and 51 controls. We classified genes expressed in the prefrontal cortex (analyzed by RNA-seq) into several modules by weighted gene co-expression network analysis (WGCNA) and examined the correlation between module expression and clinical characteristics. In addition, we calculated the polygenic risk score (PRS) for schizophrenia from Japanese genome-wide association studies, and investigated the association between the identified gene modules and PRS to evaluate whether genetic background affected gene expression. Finally, we conducted pathway analysis and upstream analysis using Ingenuity Pathway Analysis to clarify the functions and upstream regulators of symptom-related gene modules. As a result, three gene modules generated by WGCNA were significantly correlated with clinical characteristics, and one of these showed a significant association with PRS. Genes belonging to the transcriptional module associated with PRS significantly overlapped with signaling pathways of multiple sclerosis, neuroinflammation, and opioid use, suggesting that these pathways may also be profoundly implicated in schizophrenia. Upstream analysis indicated that genes in the detected module were profoundly regulated by lipopolysaccharides and CREB. This study identified schizophrenia symptom-related gene sets and their upstream regulators, revealing aspects of the pathophysiology of schizophrenia and identifying potential therapeutic targets.

## Introduction

Schizophrenia is a multifactorial disorder with high heritability [1], and most medications available or in clinical trials have been developed based on the dopamine and glutamate hypothesis [2–4]. However, current antipsychotic medications do not alleviate all symptoms and often have serious side effects [5]. As current drug discovery strategies have yielded only eight drugs to gain approval from the Food and Drug Administration in 15 years [4], new perspectives are needed to develop therapeutic drugs.

Large-scale genome-wide association studies (GWAS) have repeatedly been conducted to elucidate the complete genomic architecture of schizophrenia and have identified many single nucleotide polymorphisms (SNPs) that affect the risk of schizophrenia [6, 7]. As the effect sizes of each variant are small or negligible, the polygenic risk score (PRS) may be useful to clarify the etiology of schizophrenia [8, 9]. PRS can quantify an individual’s risk of schizophrenia by summing the weighted effect sizes of all risk and protective SNPs from the discovery GWAS and has been effectively used in basic translational and clinical medical research. PRS-based analyses revealed genetic similarities between schizophrenia and other neuropsychiatric diseases such as bipolar disorder and major depression [10, 11], as well as a significant association between PRS and clinical information, such as schizophrenia symptoms [12, 13] or the effects of antipsychotics [14].

Although PRS has achieved these goals, various issues remain to be resolved. In one study, PRS explained only 7% of the schizophrenia onset risk in the general population [7], which limits its predictive capabilities for estimating schizophrenia onset [15]. Another study reported that PRS could not predict psychotic symptoms in the general population during adolescence [16]. Furthermore, PRS failed to improve the performance of the conventional predictive model for symptom outcomes in two cohorts of patients with schizophrenia and related psychotic disorders [17]. Thus, as with similar schizophrenia research, the gap between PRS and clinical matter in schizophrenia remains large. In addition, attempts to identify the causative molecules of schizophrenia by PRS have not yet been fully conducted. One previous study examined the effects of PRS on transcripts of the dorsolateral prefrontal cortex [18], but failed to identify a single gene significantly affected by PRS. This may be because the interaction of multiple genes results in schizophrenia symptoms, and the risk of schizophrenia is similarly affected by multiple genetic variants [19].

Based on these limitations of PRS, the newer omnigenic model may explain the onset of schizophrenia more accurately [20–22]. The polygenic model (represented by PRS) only considers genes that show a significant association with the target phenotype, whereas the omnigenic model considers core genes and peripheral genes. A relatively small number of core genes are regulated by a larger number of peripheral genes, and the interaction between these two types forms the clinical phenotype. This omnigenic model is still not sufficient to examine the association between single gene expression and phenotype; therefore, we must examine the association between gene sets and the phenotype.

In this context, weighted gene co-expression network analysis (WGCNA) is considered an effective methodological approach [23]. WGCNA can identify interacting gene sets by dividing genes with similar expression into modules. A previous study attempted to verify the omnigenic model using WGCNA, and reported that the effects of all genes (core and peripheral) should be considered when conducting network analysis [24].

In addition, several previous studies using WGCNA have identified gene sets associated with schizophrenia [25–29]. For example, Radulescu et al. [25] identified gene modules that were correlated with schizophrenia and PRS and revealed that these gene sets were enriched in schizophrenia-related genes reported by the Psychiatric Genomic Consortium. They concluded that WGCNA is a promising tool for comprehensively examining genes involved in the pathogenesis of schizophrenia and for identifying candidate therapeutic targets. However, only a few studies have examined gene clusters associated with schizophrenia symptoms. To the best of our knowledge, study by Zhang et al. [29] is the only such study that revealed an association between abnormal psychomotor behavior and a gene set related to immune pathways, but they analyzed differentially expressed genes in peripheral blood leukocytes. Therefore, no study has evaluated the link between transcriptome data from the human brain and schizophrenia symptoms.

The present study aims to classify transcriptome data from the postmortem prefrontal cortex (PFC) into gene modules using WGCNA and to identify gene modules correlated with schizophrenia symptoms. In addition, it will examine the connection between identified gene modules and PRS (calculated from Japanese GWAS) to evaluate whether genetic background affects gene expression. Using Ingenuity Pathway Analysis (IPA) to conduct pathway and upstream analysis, we aim to reveal the function and regulators of these symptom-related genes.

## Patients and methods

### Patients

Postmortem brain samples from 26 patients with schizophrenia and 51 control patients were obtained from the Fukushima Brain Bank of the Department of Neuropsychiatry, School of Medicine, Fukushima Medical University, and the Brain Research Institute, Niigata University. This study was approved by the ethics committees of Fukushima Medical University, Niigata University, and the Tohoku University Graduate School of Medicine. All procedures were performed after written informed consent was obtained from the next-of-kin. The demographic information of patients in each group is shown in Table 1. Criteria from the fourth edition of the Diagnostic and Statistical Manual of Mental Disorders (DSM-IV) were used to diagnose schizophrenia. The Diagnostic Instrument for Brain Studies (DIBS) was used to evaluate the antemortem symptoms of each patient with schizophrenia 3 months before their death [30–33]. We classified each item of antemortem symptoms of DIBS into three subscales: positive symptoms, negative symptoms, and general psychopathology, according to the Positive and Negative Syndrome Scale [34]. Each score when the total DIBS score was maximum and when three months before death was used. For patients with schizophrenia. The daily dosage of antipsychotics for patients with schizophrenia three months before death is described as the chlorpromazine equivalent dose (CP eq). To evaluate their responsiveness to antipsychotics, we calculated the ratio of relative responsiveness to daily CP eq as the treatment resistance score. Relative responsiveness was calculated as the percentage of improvement in positive symptom score when compared to the score at its most severe.

$$({\rm{treatment}}\,{\rm{resistance}}\,{\rm{score}}) = \frac{{({\rm{change}}\;{\rm{rate}}\;{\rm{of}}\;{\rm{the}}\;{\rm{positive}}\;{\rm{symptoms}}\;{\rm{score}})}}{{({\rm{daily}}\;{\rm{CP}}\;{\rm{eq}}\;{\rm{at}}\;{\rm{three}}\;{\rm{months}}\;{\rm{before}}\;{\rm{death}})}}$$

$$({\rm{change}}\;{\rm{rate}}\;{\rm{of}}\;{\rm{the}}\;{\rm{positive}}\;{\rm{symptoms}}\;{\rm{score}}) = \frac{{\left( {{\rm{improvement}}\;{\rm{of}}\;{\rm{the}}\;{\rm{positive}}\;{\rm{symptoms}}\;{\rm{score}}\;{\rm{from}}\;{\rm{the}}\;{\rm{most}}\;{\rm{severe}}} \right)}}{{({\rm{most}}\;{\rm{severe}}\;{\rm{positive}}\;{\rm{symptoms}}\;{\rm{score}})}}$$

**Table 1:** Demographic data of all patients.

	Schizophrenia	Control	*p*-value
Number of samples	26	51	
Age at death^a^	68.8 ± 11.3	64.1 ± 15.1	0.136^b^
PMI^a^	16.7 ± 11.3	11.0 ± 15.5	0.075^b^
Sex			0.886^c^
Male	17	31	
Female	9	20	

^a^Data are reported as mean ± standard deviation.

^b^Welch’s *t* test.

^c^Chi square-test.

Genotyping and PRS calculations were performed on 24 patients and 51 controls (Supplementary Table 1), and RNA sequencing (RNA-seq) and gene expression analyses were performed on 25 patients and 21 controls (Supplementary Table 2). Among the 25 patients with measured gene expression, 12 patients fulfilled all items, 11 patients had DIBS scores but no available CP eq or treatment resistance scores, and 2 patients did not have an available DIBS score, CP eq, treatment resistance score, and pH. Among the 21 controls, 8 subjects were fulfilled all items and 13 patients were not available for pH.

### Genotyping and imputation

Genomic DNA was extracted from the frozen cerebellum or occipital cortex, and genotypes were determined using Infinium Human Exome-12 v1.2 and HumanCoreExome-24 v1.0 Beadchip on an iScan system (Illumina, Tokyo, Japan), as described in our previous studies [31, 32, 35]. Genotyping was conducted in 24 patients with schizophrenia and 48 controls. We used the following criteria to select SNPs: (1) in the autosomal region, (2) with a call rate > 90%, and 3) not duplicated or ambiguous. Ultimately, 217,405 SNPs were included in imputation. Genotype imputation was performed using the Michigan imputation server (https://imputationserver.sph.umich.edu) [36] with the 1000 Genomes Project Phase 3 dataset of East Asian ancestry [37] as a reference panel. After imputation, SNPs with low imputation quality (*R*^2^ < 0.2) were excluded, leaving 10,256,044 SNPs.

### PRS calculation

Quality checks of SNPs and PRS were conducted using PLINK v1.9 (http://www.cog-genomics.org/plink/1.9) [38] and PRSice-2 [39]. After imputation, SNPs were excluded if: 1) the minor allele frequency was low (< 0.001), and 2) they deviated from Hardy-Weinberg equilibrium (*p* < 1.0 × 10^–5^). In total, 7,596,758 SNPs were identified. Next, SNPs were pruned based on a pairwise r^*2*^ threshold of 0.25 and a window size of 200 SNPs, leaving 443,419 SNPs. As no samples in this analysis showed high relatedness (>0.125), none were considered to be related. PRS was calculated with Japanese samples using publicly available GWAS from the NBDC Human Database by the Japan Science and Technology Agency (hum0197.v3.gwas.v1), which integrates the genomic information of 179,000 patients with 215 phenotypes (including schizophrenia) as discovery GWAS [40]. The significance threshold (*P*_T cutoff_) for SNP inclusion was determined by the *p* value at which the coefficient of determination (*R*^2^) predicted the onset of schizophrenia.

### mRNA expression

Total RNA was isolated from the PFC of the frozen brain using an AllPrep DNA/RNA Mini Kit (Qiagen, Tokyo, Japan). RNA purity was evaluated by the RNA integrity number (RIN), which was determined using an Agilent 2200 TapeStation (Agilent, Santa Clara, CA, USA). The poly (A) fraction was isolated from total RNA, followed by its fragmentation. Next, base pairs (bp) of double-stranded (ds) complementary DNA (cDNA) were reverse-transcribed from the fragmented mRNA. The ds-cDNA fragments were processed for adapter ligation, size selection (for 200 bp inserts), and amplification to generate cDNA libraries. The prepared libraries were subjected to paired-end 2 × 101 bp sequencing on the HiSeq 4000 platform, using the HiSeq 3000/4000 SBS Kit (Illumina, Tokyo, Japan). Data from 25 patients with schizophrenia and 21 controls in the PFC were obtained. Genes with low expression were filtered using the egdeR software package [41]. No replicated experiment was conducted.

### Weighted gene co-expression analysis (WGCNA)

We conducted WGCNA on 15,938 coding-RNA expressed in the PFC of patients with schizophrenia and controls using R statistical analysis software (version 4.1.1) and its WGCNA package [23]. The topological overlap matrix (TOM) was calculated using a soft-threshold power 12, and a signed network was constructed. Furthermore, 1−TOM was used as a distance matrix for hierarchical clustering, and all transcripts were assigned to modules. The minimum module size was set at 200. The modules were represented as different colors (e.g., blue, yellow, red).

### Fold-changes of gene expression

We calculated log2 fold-changes of gene expression between controls and patients with schizophrenia using R and the egdeR package, and these calculations were used in IPA analysis. We set the following as covariates: sex, age, PMI (postmortem interval), and RIN. As the sample size was relatively small, continuous covariates were factored into bins, as per a previous study [42]. Age was categorized by groups of 10 years, PMI was categorized by intervals of 5 h, and RIN was categorized by units of 1.

### Statistical analysis

Covariates in demographic data were compared between controls and patients with schizophrenia; Chi square tests were used for categorical variables (sex) and Welch’s *t*-tests were used for continuous variables (age, PMI, and pH). Welch’s test was also used to compare the PRS calculated for each sample between controls and patients with schizophrenia. We investigated the correlations between clinical characteristics of schizophrenia including symptoms and treatment responsiveness and the module eigengene, defined as the first principal component of the expression matrix in each module classified by WGCNA. To analyze the correlations between gene modules and antemortem symptoms, we separately conducted canonical pathway analysis and upstream regulator analysis in IPA. All analyses used *p* < 0.05 to indicate statistical significance.

## Results

### PRS calculation

**Fig. 1:**
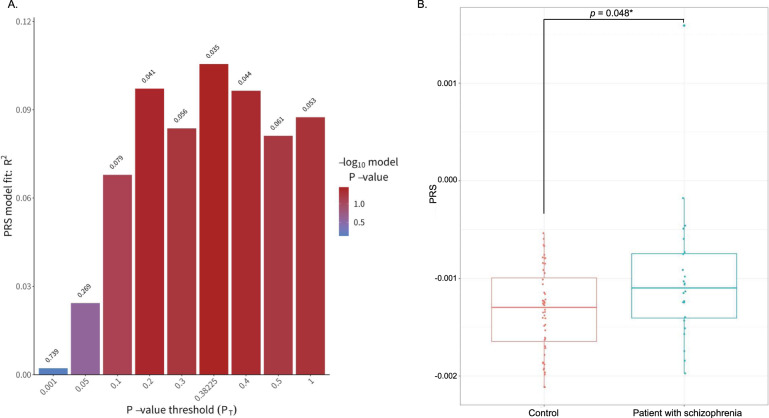
Results of PRS calculation. **A** A bar plot shows which *p*-value threshold matches the PRS most closely. When the *p* value threshold is 0.382, PRS explains the largest variations of risk of schizophrenia greatest with *R*^2^ = 0.106. **B** PRS was significantly higher in patients with schizophrenia than controls (*p* = 0.048).

We first investigated the effect of the PRS (calculated at different *P*_T cutoff_ values) on the risk of schizophrenia (Fig. 1A). As the maximum of *R*^2^ = 0.106 when *p* = 0.382, we used *P*_T cutoff_ = 0.382 for the following analysis. The PRS was significantly higher in patients with schizophrenia than in controls (Fig. 1B, *p* = 0.048).

### Weighted gene co-expression analysis (WGCNA)

**Fig. 2:**
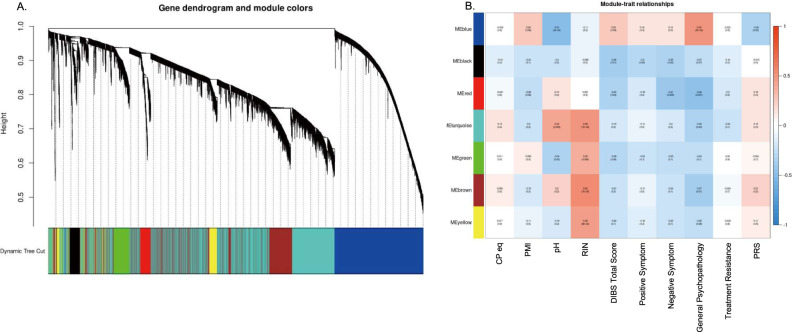
Results of gene clustering and gene-clinical characteristics correlation. **A** Genes were classified into seven modules by WGCNA using 1−TOM. **B** A heatmap shows correlation between module eigengene of each cluster and cofounders, antemortem schizophrenia symptoms, and PRS. The *p* value is shown in parentheses.

Network construction using WGCNA classified 15,938 transcripts into seven separate modules and dendrogram was produced using 1−TOM (Fig. 2A), represented by different colors along the x-axis. To identify the gene modules that correlate with clinical characteristics and PRS, we investigated module-trait associations (Fig. 2B). Blue gene module expression (*n* = 2823) was significantly correlated with the general psychopathology score (*r* = 0.49, *p* = 5.0 × 10^–4^) and PRS (*r* = −0.35, *p* = 0.02). Black gene module expression (*n* = 449) was significantly correlated with the DIBS total score (*r* = −0.36, *p* = 0.01), positive symptom score (*r* = −0.30, *p* = 0.05), and negative symptom score (*r* = −0.35, *p* = 0.02). Red gene module expression (*n* = 504) was significantly correlated with the DIBS total score (*r* = −0.33, *p* = 0.03), negative symptom score (*r* = −0.41, *p* = 0.005), and general psychopathology score (*r* = −0.46, *p* = 0.001). The genes assigned to these three modules are listed in Supplementary Table 3. Although the turquoise (*n* = 6652) and brown (*n* = 3823) gene modules also showed significant correlations with general psychopathology scores, these two modules were excluded from further analysis because they were significantly affected by RIN, a measurement of the quality of mRNA (turquoise gene module: *r* = 0.48, *p* = 7.0 × 10^–4^, brown gene module: *r* = 0.55, *p* = 7.0 × 10^–5^), while no other modules were affected by RIN. Blue gene module also showed a significant correlation with pH (*r* = −0.51, *p* = 3.0 × 10^–4^), but it was included in further analysis since tissue pH have been reported to be related with pathophysiology of schizophrenia [43].

### Ingenuity pathway analysis (IPA)

**Fig. 3:**
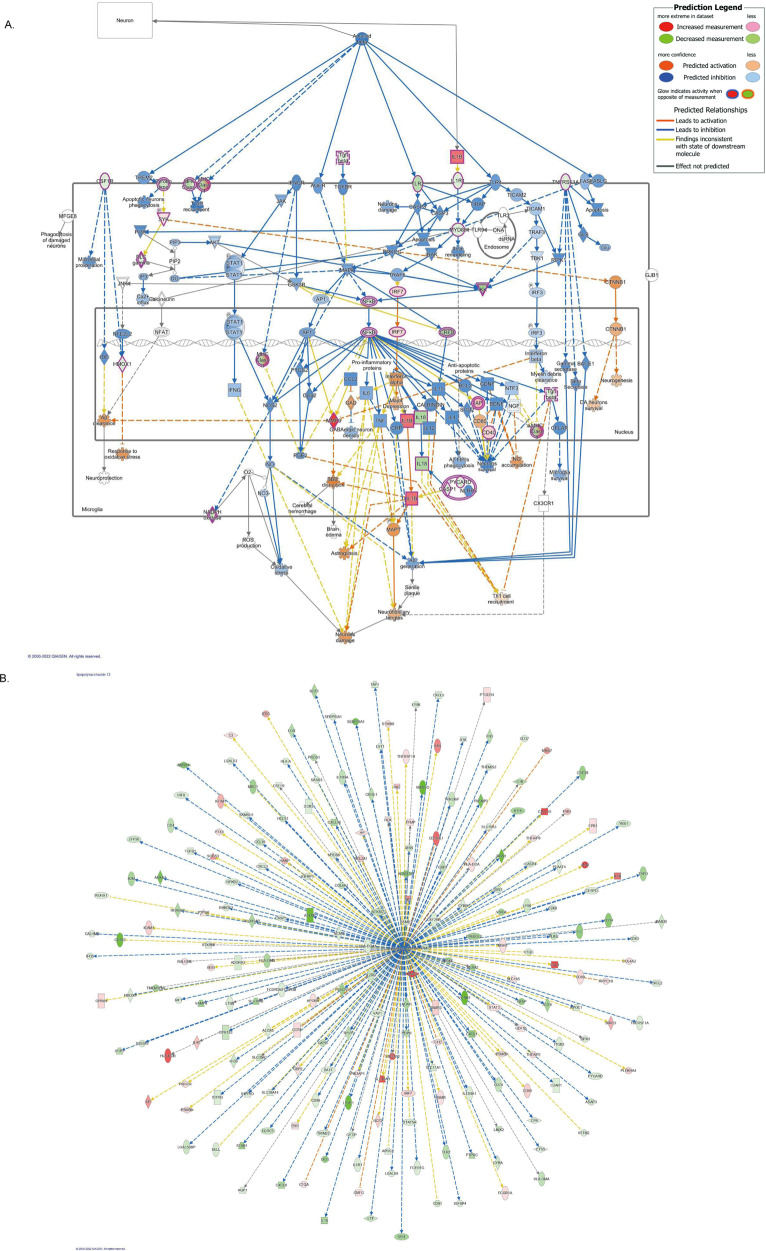
Bioinformatic analysis of black gene module by IPA. **A** A canonical pathway of the neuroinflammation signaling pathway is downregulated in patients with schizophrenia. Genes highlighted in pink are included in the black module; red represents upregulated expression while green represents downregulated expression. Genes noted in orange were predicted to be upregulated, while those noted in blue were predicted to be downregulated. The orange line represents the expression state of consistent activation between the upstream regulator and the gene. The blue line represents the expression state of consistent inhibition between the upstream regulator and the gene. The yellow line represents an inconsistent relationship between the upstream regulator and the gene. The gray line represents no prediction information related to the expression status. **B** LPS is an upstream regulator that modulates gene expression in the black module. Genes noted in red were upregulated, while those noted in green were downregulated. Line colors have the same meaning as in (**A**).

**Fig. 4:**
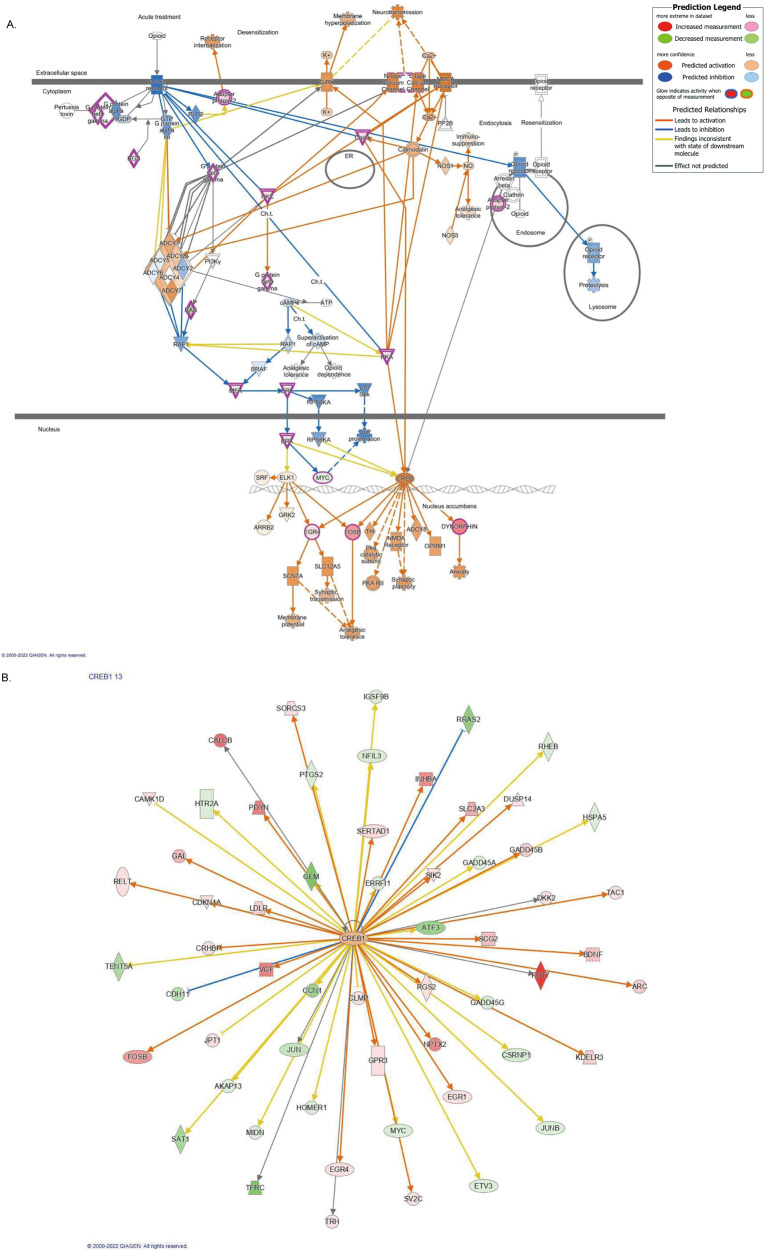
Bioinformatic analysis of red gene module by IPA. **A** A canonical pathway of opioid signaling pathway is upregulated in patients with schizophrenia. Genes highlighted in pink are included in the red module; red represents upregulated expression, and green represents downregulated expression. Genes noted in orange were predicted to be upregulated, while those noted in blue were predicted to be downregulated. The orange line represents the expression state of consistent activation between the upstream regulator and the gene. The blue line represents the expression state of consistent inhibition between the upstream regulator and the gene. The yellow line represents an inconsistent relationship between the upstream regulator and the gene. The gray line represents no prediction information related to the expression status. **B** CREB is an upstream regulator that modulates gene expression in the red module. Genes noted in red were upregulated, while those noted in green were downregulated. Line colors have the same meaning as in (**A**).

To further examine gene modules that were significantly correlated with clinical characteristics, we conducted canonical pathway analysis and upstream regulator analysis on genes in the blue, black, and red gene modules using IPA. The blue gene module showed significant overlap with the Multiple Sclerosis (MS) signaling pathway (*p* = 1.3 × 10^–5^). Ponesimod (a drug used to treat MS) was a predicted upstream factor that significantly regulated genes in the blue module (*p* = 9.9 × 10^–7^). The black gene module showed a significant overlap with the neuroinflammation signaling pathway (*p* = 2.3 × 10^–29^). Lipopolysaccharide (LPS) was a predicted upstream factor that significantly regulated genes in the black module (*p* = 2.3 × 10^–29^). IPA analysis of the black gene module also showed that the neuroinflammation signaling pathway was suppressed in patients with schizophrenia (Fig. 3A). Figure 3B shows the effect of LPS on downstream genes in the black module. The red gene module showed a significant overlap with the opioid signaling pathway (*p* = 8.8 × 10^–6^). Cyclic AMP responsive element binding protein (CREB) was a predicted upstream factor that significantly regulated genes in the red module (*p* = 2.3 × 10^–29^). CREB activation was regulated by N-methyl-D-aspartate (NMDA) and opioid receptors and was predicted to be highly activated in patients with schizophrenia (Fig. 4A). Figure 4B shows the effects of CREB on its downstream genes in the red module. The results of the canonical pathway analysis and upstream regulator analysis of the three gene modules are reported in entirety in Supplementary Tables 4 and 5.

## Discussion

In the current study, we examined 26 patients with schizophrenia and 51 healthy controls. We analyzed transcriptome data from the PFC to identify schizophrenia-related genes. Using WGCNA, we classified genes into seven gene modules that showed similar differential expression patterns in the PFC between schizophrenic and control groups and investigated their association with clinical characteristics. In addition, we evaluated the effects of genetic factors by calculating the PRS for schizophrenia. As a result, we identified three gene modules that were significantly linked to schizophrenia symptoms, and one also showed a significant relationship with the PRS. Furthermore, we examined their functions and upstream regulators using IPA to identify biological systems that are significantly affected by the pathogenesis of schizophrenia as well as potential therapeutic targets. This is the first known study to identify schizophrenia symptom-related gene modules by associating human brain gene sets (generated by WGCNA) with schizophrenia symptoms.

**Fig. 5:**
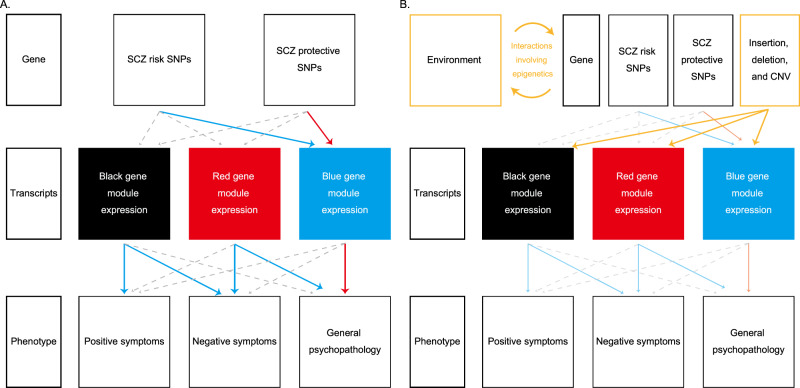
Overview of the relationship between genes, gene modules and symptoms in this study. **A** A graphic model which represents the association of genes, transcripts, and phenotype. Genetic factors associated with PRS regulate transcripts, and transcripts modules define the clinical phenotype. The red and blue arrows represent the significant upregulation and downregulation, respectively. The gray arrows represent non-significant regulation. **B** A hypothetical model which represents how other genetic factors (insertion, deletion, and copy number variants) and environmental factors may contribute to aberrant expression of transcripts. The yellow arrows represent the potential regulation by these uninvestigated factors.

The relationship revealed in this study between genetic factors, transcriptome, and phenotype is shown in Fig. 5A as a three-layer structure. In this model, expression level of each gene fluctuates depending on genetic variants, and the resulting transcripts form the clinical phenotype. In particular, the influence of schizophrenia-associated SNPs was incorporated into the blue gene module. In addition, transcripts of the black, red, and blue gene modules may be causative for each type of schizophrenia symptom. Although the three gene modules showed close links with antemortem symptoms, only the blue module was significantly associated with the PRS. IPA analysis elucidated the characteristics of blue, black, and red gene modules and their relationship to schizophrenia symptoms.

The blue module was strongly associated with MS, and Ponesimod (a therapeutic agent for MS) was also identified as an upstream regulator. Comorbidity and similarity between MS and schizophrenia have been previously researched. A review by Murphy et al. reported that patients with MS frequently presented with psychiatric symptoms and emphasized the importance of considering this comorbidity during differential diagnosis [44], especially as some patients with MS initially present only psychiatric symptoms, including auditory hallucinations, persecutory delusions, and cognitive impairment [45]. Despite this difficulty, recognizing psychotic comorbidities of conditions like MS is essential for effective treatment and improving the quality of life for patients. As some typical antipsychotics may cause extra-pyramidal side effects (also seen in MS), it must be noted that patients with both MS and psychosis should be prescribed atypical antipsychotics, which have fewer side effects [46]. Moreover, several reports have suggested a relationship between these two diseases from a genetic and transcriptomic perspective [47, 48]. For example, Ahangari et al. analyzed a GWAS dataset of over 20,000 individuals and revealed new loci associated with both MS and schizophrenia [47]. Our current results strongly support the idea that these two diseases share common causative gene sets. As this module showed a significant association with the general psychopathology score, it is possible that schizophrenia-related molecules that are vulnerable to cognitive and affective dysfunction may also be associated with MS. The commonality of these diseases remains unclear and should be addressed by future studies.

In addition, the blue gene module showed a significant correlation with tissue pH. Since tissue pH has been reported to be affected in an agonal state [49], it is possible that the expression of the blue gene module have been affected by the agonal state. However, there are several reports that tissue pH was related to pathophysiology itself of schizophrenia [43]. For example, brains of patients with schizophrenia exhibited significantly lower tissue pH even though several covariates such as age, PMI, and intake of antipsychotics were considered [50]. Therefore, it is undeniable that the blue gene module is closely associated with pathophysiology of schizophrenia and it is likely that the expression of the blue gene module has some relationship with pathological downregulation of tissue pH in patients with schizophrenia.

The black module was associated with genes related to inflammation, and the following analysis suggested that patients with low expression of these genes tended to report more severe schizophrenia symptoms. As this module showed a significant association with positive and negative symptom scores, the inflammation-related gene module may be related to these symptoms. Several previous studies have demonstrated a link between inflammation and symptoms of schizophrenia [51–53]. Li et al. reported that TLR4/NF-κB/IL-1β signaling (an essential pathway of innate immunity) is less responsive to LPS stimulation in patients with schizophrenia [51], which is consistent with our results that the downregulation of gene sets controlled by LPS is associated with severity of schizophrenia symptoms. In addition, Zhang et al. [29] conducted WGCNA on RNA-seq data of peripheral blood leukocytes and showed that the expression of gene sets in the NF-κB signaling pathway was closely linked to abnormal psychomotor behavior. Our findings support these results and strongly suggest that gene sets affected by LPS may be essential in the pathophysiology of schizophrenia.

The red module is a gene set regulated by CREB, a transcription factor known to be linked to schizophrenia. Previous studies demonstrated that CREB was overexpressed in the PFC of patients with schizophrenia [54] and SNPs in genes rs2709370 and rs6785 affected the risk of schizophrenia [55]. Moreover, rs2709370 was significantly associated with hippocampal structure and function [56], which were associated with schizophrenia [57, 58]. Our results suggest that CREB signaling is activated in patients with schizophrenia, and the subsequent activation of downstream genes of CREB may affect the severity of negative symptoms, cognitive dysfunction, and affective dysfunction. CREB regulates some schizophrenia symptom-related gene sets and may be a promising therapeutic target, despite the lengthy interval between the detection of candidate molecules and the utilization of novel therapeutic agents. Oligonucleotides may be potential agents for controlling CREB activation. Regulation of gene expression using antisense oligonucleotides complementary to the target transcripts has been developed for the clinical treatment of neuropsychiatric disorders, such as major depressive disorder and Parkinson’s disease (PD) [59, 60]. For example, leucine-rich repeat kinase 2 (LRRK2) is a causative gene of PD [61] and an important therapeutic target for PD because its antisense oligonucleotide has been shown to decrease LRRK2 protein levels [62]. The LRRK2 antisense oligonucleotide BIIB094 is currently in phase 1 clinical trial (NCT03976349). Oligonucleotides have not been fully explored for schizophrenia. Based on the schizophrenia symptom-related gene modules and their upstream regulators identified in the current study, oligonucleotides should be increasingly paid attention as therapeutic agents for schizophrenia.

In contrast, only the blue module showed an association with the PRS, although several gene modules were associated with schizophrenia symptoms. This means that the conventional polygenic model reflecting the risk of schizophrenia only explains a limited number of gene sets related to schizophrenia. Although the expression of the blue module was positively correlated with general psychopathology score, it was negatively correlated with PRS. This unexpected relationship among PRS, transcripts, and clinical phenotypes suggests that patients with higher PRS scores show less cognitive and affective dysfunction caused by the gene set of the blue module. Our results did not support a previous finding that higher PRS is associated with lower cognitive function [12].

As conventional PRS did not fully explain the aberrant expression of schizophrenia symptom-related gene modules, factors other than the influence of PRS must explain gene expression patterns in these patients. Examples include genetic factors other than SNPs, as some studies have reported that specific insertions, deletions, and copy number variations (CNV) were associated with schizophrenia [63, 64]. 22q11.2 deletion is considered the most well-known CNV related to schizophrenia [65]. Actually, some genes in 22q11.2 region such as GP1BB and TBX1 are included in the blue gene module and GNB1L and ZNF74 are included in the red gene module. Thus, this CNV region may have some relationship with the current identified gene modules. This holds true to other CNV regions like 3q29, which is also one of the well-known [66], because several genes in 3q29 deletion region such as SMCO1. WDR53, PIGX, and NCBP2 are also included in the blue gene module and TFRC and PAK2 are included in the red gene module. Therefore, these genetic factors, as well as environmental factors such as maternal infection and prenatal malnutrition, should be measured when evaluating the risk of schizophrenia.

It is crucial to focus on epigenetic changes when examining environmental factors. Several studies have revealed a relationship between schizophrenia and epigenetic profiling of specific genes [67, 68]. For example, a systematic review by Lockwood et al. [68] included 17 studies that investigated the association between epigenetics and first-episode psychosis. Among these, two studies reported that the promoter region of GRIN2B, a gene encoding subunit of the NMDA receptor, is hypomethylated [69, 70]. Thus, it is likely that the expression of genes associated with schizophrenia is altered by epigenetic mechanisms reflecting environmental factors, and further postmortem brain analysis investigating epigenetic profiling is required.

Additional analyses will be essential to evaluate the other genetic and environmental factors that influence the expression of the schizophrenia-related genes identified in this study. Although the conventional polygenic model is useful for evaluating the risk of schizophrenia to some extent, its association with gene expression remains insufficient. Further analysis of schizophrenia symptom-related genes should be conducted, including other genetic factors (such as insertion, deletion, and CNV) and epigenic profiling representing environmental factors, which would reveal the detailed pathophysiology of schizophrenia (Fig. 5B). Links between schizophrenia and MS, neuroinflammation, and opioid use should also be explored in future studies.

This study had several limitations. First, the sample size was relatively small because of the strict inclusion criteria of this study, which required postmortem brain samples and antemortem clinical information. Second, the influence of potential confounds was not taken into account. Although we set the intake of antemortem antipsychotics, PMI, and RIN as covariates, the effects of smoking, drinking, and cause of death were not considered; therefore, these factors could have influenced the results. In particular, molecules associated with inflammation may differ by cause of death and agonal state. Therefore, further studies are required to address these limitations.

In conclusion, when the genes were grouped according to their expression patterns, the current study identified three gene sets that may be related to schizophrenia symptoms and further revealed that one of them was significantly associated with PRS in schizophrenia. This study is the first to investigate the association between genetic risk and transcriptional profiles in the brains of patients with schizophrenia at a genome-wide level, with the aim of understanding the clinical manifestations of the disease. The network of genes, transcripts, and schizophrenia symptoms reported in this study will be useful for elucidating the pathophysiology of schizophrenia and searching for novel and promising therapeutic targets. These findings are an important stepping stone toward understanding each symptom of schizophrenia at the molecular level.

## Supplementary information

Supplementary Table
